# Effects of red meat taxes and warning labels on food groups selected in a randomized controlled trial

**DOI:** 10.1186/s12966-024-01584-9

**Published:** 2024-04-15

**Authors:** Amelia Willits-Smith, Lindsey Smith Taillie, Lindsay M. Jaacks, Sarah M. Frank, Anna H. Grummon

**Affiliations:** 1 Carolina Population Center, University of North Carolina at Chapel Hill, 27516 Chapel Hill, NC USA; 2 Department of Nutrition, Gillings School of Global Public Health, University of North Carolina at Chapel Hill, 27516 Chapel Hill, NC USA; 3 Global Academy of Agriculture and Food Systems, The University of Edinburgh, Midlothian, UK; 4 Department of Pediatrics, Stanford University School of Medicine, 3145 Porter Drive, A103, 94034 Palo Alto, CA USA; 5 Department of Health Policy, Stanford University School of Medicine, 94305 Stanford, CA USA

**Keywords:** Red meat, Processed meat, Health, Sustainability, Warning label, Tax, Food policy, Food purchases, Randomized trial

## Abstract

**Background:**

High consumption of red and processed meat contributes to both health and environmental harms. Warning labels and taxes for red meat reduce selection of red meat overall, but little is known about how these potential policies affect purchases of subcategories of red meat (e.g., processed versus unprocessed) or of non-red-meat foods (e.g., cheese, pulses) relevant to health and environmental outcomes. This study examined consumer responses to warning labels and taxes for red meat in a randomized controlled trial.

**Methods:**

In October 2021, we recruited 3,518 US adults to complete a shopping task in a naturalistic online grocery store. Participants were randomly assigned to one of four arms: control (no warning labels or tax), warning labels only (health and environmental warning labels appeared next to products containing red meat), tax only (prices of products containing red meat were increased 30%) or combined warning labels + tax. Participants selected items to hypothetically purchase, which we categorized into food groups based on the presence of animal- and plant-source ingredients (e.g., beef, eggs, pulses), meat processing level (e.g., processed pork versus unprocessed pork), and meat species (e.g., beef versus pork). We assessed the effects of the warning labels and tax on selections from each food group.

**Results:**

Compared to control, all three interventions led participants to select fewer items with processed meat (driven by reductions in processed pork) and (for the tax and warning labels + tax interventions only) fewer items with unprocessed meat (driven by reductions in unprocessed beef). All three interventions also led participants to select more items containing cheese, while only the combined warning labels + tax intervention led participants to select more items containing processed poultry. Except for an increase in selection of pulses in the tax arm, the interventions did not affect selections of fish or seafood (processed or unprocessed), eggs, or plant-based items (pulses, nuts & seeds, tofu, meat mimics, grains & potatoes, vegetables).

**Conclusions:**

Policies to reduce red meat consumption are also likely to affect consumption of other types of foods that are relevant to both health and environmental outcomes.

**Trial registration:**

NCT04716010 on www.clinicaltrials.gov.

**Supplementary Information:**

The online version contains supplementary material available at 10.1186/s12966-024-01584-9.

## Introduction

Every year, the average US consumer eats more than 84 pounds (38 kg) of red meat (i.e., beef, veal, pork, lamb, or mutton) [1]. By contrast, the EAT-Lancet reference diet recommends eating less than 22.5 pounds (10.2 kg) per year [2], and the US Dietary Guidelines for Americans recommend limiting red and processed meat [3]. High consumption of red meat poses risks to both human health and environmental sustainability. Although red meat contains protein, heme iron, zinc, B vitamins, and other nutrients [4, 5], a growing body of evidence indicates that individuals who eat high levels of red meat are more likely to develop diet-related chronic diseases including cardiovascular disease [6–8], type 2 diabetes [7, 9], and some types of cancer [10–14]. Further, producing red meat contributes to a range of environmental harms including greenhouse gas emissions [15–19], air and water pollution [7, 15], deforestation [20, 21], and biodiversity loss [2, 22]. Reducing the amount of red meat Americans eat could therefore simultaneously reduce rates of diet-related chronic diseases *and* lessen the negative environmental impacts of food production.

Policymakers are increasingly interested in adopting policies to address the health and environmental harms of red meat production and consumption [23, 24]. One such potential policy is requiring warning labels on product packaging that inform consumers about the health and environmental harms of red meat [24–27], similar to the warning labels required on alcohol in more than 40 countries [28]. Another promising policy is raising the price of red meat through taxes [24, 25], similar to taxes levied on other unhealthy products like alcohol and sugary drinks [29–31]. Several recent empirical and modeling studies suggest that warning labels and taxes could meaningfully reduce selection and purchases of red meat [32–40].

What is less well studied is how warning labels and taxes for red meat affect purchases across the range of foods consumers buy, including purchases of non-red-meat products that could be substitutes for or complements to red meat. This is an important gap because the health and environmental benefits of warning labels and taxes depend on consumers’ overall food purchasing patterns, rather than on changes in red meat purchases only. First, the health and environmental benefits of red meat warning labels and taxes depend on the *food groups* consumers buy instead of red meat in response to these policies. For example, we would expect both health and environmental benefits from warning labels and taxes on red meat if these policies led consumers to shift away from red meat and toward pulses, which have a generally healthy nutritional profile with low environmental impacts [17, 41, 42]. By contrast, if red meat warning labels and taxes caused consumers to shift toward meat mimic products, this would yield substantial environmental benefits [43, 44], but likely smaller health improvements, given that these products are often nutritionally similar to their meat analogues and high in sodium, among other potential nutritional concerns [45, 46]. Second, the health benefits of red meat warning labels and taxes depend on the *processing level* of any meats consumers select in response to these policies. Although both processed meat and unprocessed meat have similar environmental impacts, processed meat (especially processed red meat) is considered a stronger contributor to diet-related chronic disease risk than unprocessed meat [9, 47, 48]. Third, the environmental benefits of these policies depend on the *species* of meat consumers select because producing ruminant animals such as cattle and sheep is more environmentally harmful than producing pigs, even though beef, lamb, and pork are all considered red meat. To our knowledge, however, no randomized trials have examined purchases of non-red-meat foods or subtypes of red meat (e.g. processed versus unprocessed; pork versus beef) after implementation of red meat warning labels or taxes, so the causal effects of these policies on overall food purchasing patterns remain largely unknown.

To address these gaps, the objective of this study was to describe the effects of red meat warning labels and taxes on consumers’ selections in a randomized controlled trial. We previously published the primary results from this trial, finding that both warning labels and taxes reduced selections of red meat, with the largest reductions seen when combining the two interventions [49]. In the present study, we assessed the effect of the warning labels and tax on consumers’ entire shopping basket, including examining selections by food group (e.g., beef, pork, poultry, seafood, eggs, pulses), processing level of meat (when applicable, e.g., processed pork versus unprocessed pork), and species of meat (when applicable, e.g., beef versus pork).

## Methods

### Sample

CloudResearch (Prime Research Solutions LLC, New York) recruited a convenience sample of adults designed to approximate US age, gender, race/ethnicity, and income distributions. To be included, participants had to be at least 18 years of age, currently reside in the US, do at least half of the grocery shopping for their household, and have eaten red meat one or more times in the previous month. Of 4,158 eligible participants who began the survey, the final sample included the 3,518 (85%) participants who completed the shopping task (see Design & procedures, below).

### Setting

The trial took place in a simulated online grocery store that used data scraped from a major US food retailer. Details of the development and validation of the online store have been published previously [50, 51]. The store included more than 13,000 products and mimicked the appearance and functionality of real online grocery shopping, including allowing participants to browse, search, add items to a cart, and check out.

### Design & procedures

**Fig. 1:**
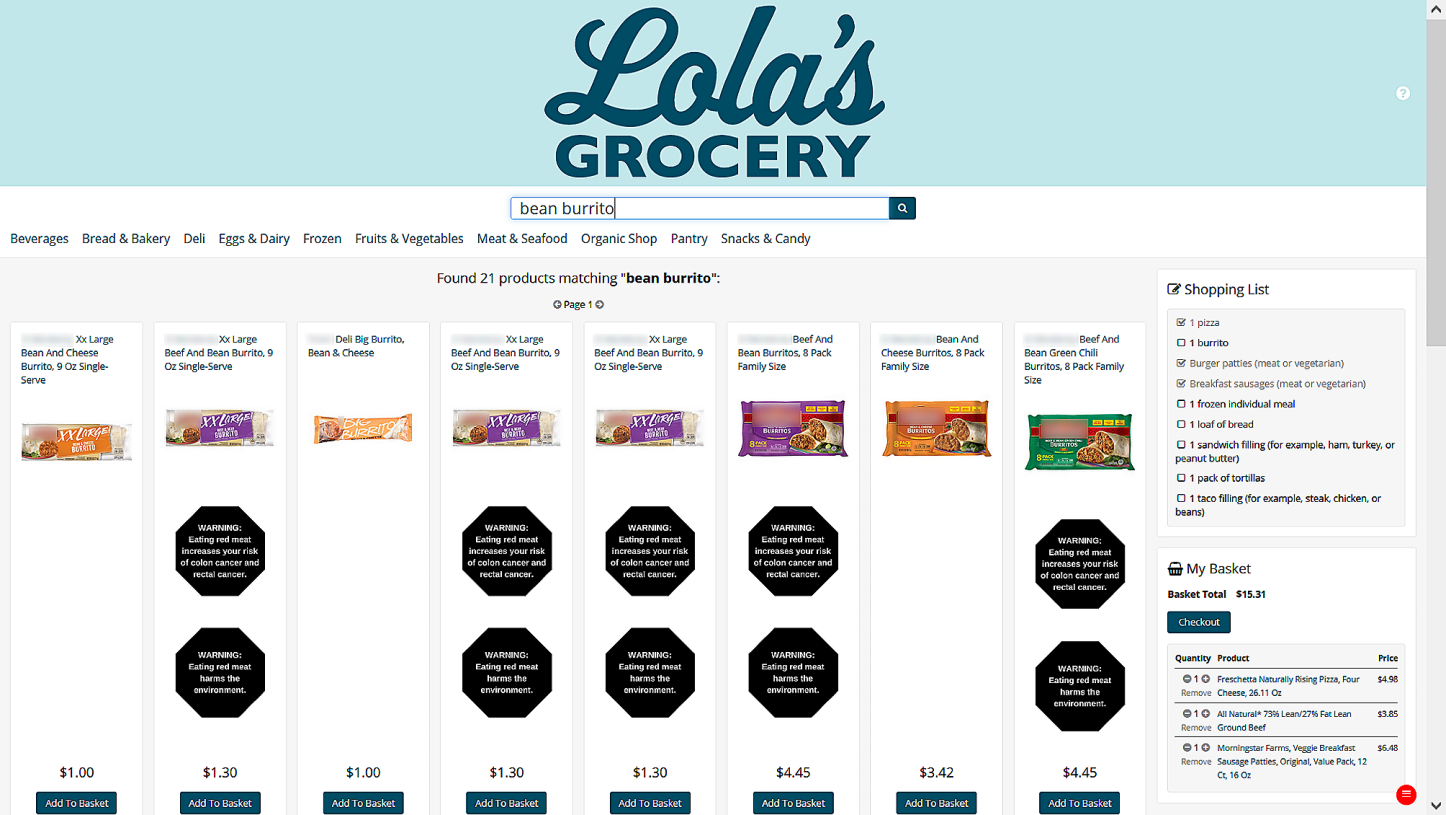
Simulated online grocery store used in randomized-controlled trial This screenshot shows an example of the store in action under the combined warning labels + tax arm. On the right-hand side is the shopping list participants are instructed to follow. In the warning labels and combined warning labels + tax arms, items with red meat display two octagonal warning labels. These labels show up in search results as well as on individual product pages. Taxes (30%) on products containing red meat are applied in the tax and combined warning labels + tax arms, but are not indicated in any way to the participant. For example, the price of a single beef and bean burrito is $1.30 in the tax or combined warning labels + tax arms (as shown here). If a participant were randomized to the control or warning labels arm, the price for the same beef and burrito would be $1.00

After providing informed consent, participants were instructed to shop in the simulated online grocery store. Participants were randomized by the survey software to one of four trial arms: control, red meat warning labels, red meat tax, or combined warning labels + tax. For participants in the warning labels or combined warning labels + tax trial arms, the store displayed health and environmental warning labels next to foods containing red meat. The two labels were used simultaneously based on the results of previous research that found that presenting both health and environmental warning labels together may be more effective than presenting either alone [26, 27]. For participants in the tax or combined warning labels + tax trial arms, the store displayed prices for foods containing red meat that were 30% higher than the control. We chose to increase prices by 30% because prior research indicates that a tax rate of this magnitude or larger would be optimal for addressing the red-meat-related health harms in the US [33]. Figure 1 shows the layout of the online store.

Participants were instructed to shop in the online store using a shopping list. The shopping list included nine items informed by previous research indicating which types of foods are the most important contributors to Americans’ intakes of red and processed meats [1]. The list asked participants to shop for: 1 pizza, 1 burrito, burger patties (meat or vegetarian), breakfast sausages (meat or vegetarian), 1 frozen individual meal, 1 loaf of bread, 1 sandwich filling (for example, ham, turkey, or peanut butter), 1 pack of tortillas, and 1 taco filling (parentheticals were included in the instructions shown to participants). To check out of the simulated online store, participants’ shopping carts needed to be within 2 items of the total number of items on the shopping list (i.e., 7–11 items).

The budget for the shopping task was $40. No payment was required. To encourage participants to select items they actually wished to purchase, the survey informed participants that they would be entered into a drawing to receive the groceries they chose along with the remainder of their shopping budget in cash. After the shopping task, participants completed an online questionnaire.

### Measures

The primary outcomes of this exploratory secondary analysis were selections of each of 18 *food groups*, operationalized as the total number of items participants selected in each food group. Since purchasing behavior in response to the warning labels or tax might differ for different types of items (e.g., when shopping for a pizza versus a sandwich filling), we also examined selections of each food group stratified by shopping list item (i.e., by *item type*).

#### Food groups

To assign products into food groups, we began by coding each product selected based on the presence or absence of different ingredients, including different meats, dairy products, eggs, pulses, tofu, meat mimics, grains & potatoes, and vegetables (for the full list of ingredients examined, see Supplemental Table 1). When a product contained meat, we additionally coded those meats for (1) whether the meat was processed or unprocessed, and (2) the species or species group of the meat (i.e., beef, pork, poultry, fish & seafood, or other ruminant animal). Processed meat was defined as “meat that has been transformed through salting, curing, fermentation, smoking, or other processes to enhance flavor or improve preservation” [14] and included bacon, sausage, deli or lunch meats, hot dogs and smoked meats. All other meat, poultry, fish and seafood were classified as unprocessed. Because nutrition facts information for packaged products does not include information on quantity of ingredients (e.g., grams of beef in a cheeseburger), we coded products as containing each ingredient if they contained any amount of that ingredient (exceptions are described in Supplemental Methods). Products could be coded as having more than one ingredient (e.g., a cheeseburger would be coded as having both beef and cheese). After coding for the presence or absence of these ingredients, we developed additional codes inductively to capture the variety of other products participants selected that were not part of the trial’s shopping list: beverages, desserts, fruit, fats, and condiments (Supplemental Table 1). These foods were excluded from analyses. Products were independently coded by two research assistants, then compared. The authors resolved any discordance.

Next, we assigned products to 18 mutually exclusive food groups based on the ingredient codes described above. We developed the food groups to approximately reflect different levels of environmental impact from producing those food groups. First, we grouped products based on the presence of any meat. Second, if the product did not contain meat, we grouped products based on the presence of dairy or eggs. Third, if the product did not contain meat, dairy, or eggs we grouped the product based on the presence of other foods that may be plausible substitutes for red meat or that were relevant to health or environmental outcomes (tofu, meat mimics, pulses, nuts & seeds, grains & potatoes, or vegetables). As an example, a cheeseburger was assigned to the unprocessed beef group, because our food grouping system prioritized the presence or absence of meat over the presence or absence of dairy, given meat’s larger environmental impacts. A bean and cheese burrito and macaroni and cheese were both assigned to the cheese group. In addition to examining these mutually exclusive food groups, we also examined selection of processed meat overall and unprocessed meat overall (regardless of species), as well as vegetarian selections overall. All food groups examined represent the presence of a particular food in that product, but do not indicate amount, and all food groups contain some mixed foods. For more detail on the assignment of food groups, see the Supplemental Methods and Supplemental Fig. 1.

#### Item type

To enable assessment of whether results differ based on item type, we also matched each product participants selected to one of the shopping list items (i.e., item type) when possible. Sandwich and taco fillings were combined into one item type, since many foods could fulfill either purpose (e.g., cheese and pulled pork could both be used in sandwiches or tacos). Details on which types of products were matched to each shopping list item are shown in Supplemental Table 2. Products that did not fit into any of the shopping list items were broadly categorized (e.g., beverages, desserts) for descriptive purposes using the inductively developed codes described above (see Supplemental Methods and Supplemental Table 3) but were not included in analyses. Although the online store required that participants select 7–11 items to check out, it did not require compliance with the shopping list. However, most participants (79%) selected at least 7 of the 8 item types, with no differences across trial arms (Supplemental Table 4). Additionally, 74–96% of participants selected an item fulfilling each of the shopping list items (depending on item, Supplemental Table 5), again with no difference by trial arm.

### Statistical analysis

Participants were analyzed in the groups to which they were randomized (intention-to-treat). We used Poisson regression to examine differences compared to the control group in (1) the selection of food groups in each trial arm, and (2) the selection of food groups in each trial arm when stratified by shopping list item. (We used Poisson regression because we analyzed counts of selections in each food group.) Analyses were corrected for multiple comparisons for each model (i.e., for each food group) using a Bonferroni-Holm correction, considering three comparisons (each trial arm compared to control). We report corrected *p*-values (i.e., *q*-values) throughout. Analyses were conducted in Stata/SE v17.0 in 2023.

## Results

### Sample characteristics

A majority of participants were women (60.4%) and non-Hispanic White (73.1%) (Table 1). About one third of participants reported having a high school diploma or less education and about two-thirds (67.5%) reported household income <$74,999 per year. Participants were relatively evenly distributed across categories of age. Almost half of participants reported eating red meat 2–3 times per week; 14.1% reported eating it at least daily. A majority of participants reported that their interest in health (61.8%) or in sustainability (73.6%) was high or moderately high.

**Table 1:** Demographic, socioeconomic, and other characteristics by trial arm (*n* = 3,518)

	Control (*n* = 887)		Warning Labels (*n* = 891)		Tax (*n* = 874)		Combined Warning Labels + Tax (*n* = 866)		Overall (*n* = 3,518)	
	*n*	%	*n*	%	*n*	%	*n*	%	*n*	%
**Gender**										
Woman	539	60.8	538	60.4	530	60.6	519	59.9	2,126	60.4
Man	343	38.7	348	39.1	342	39.1	344	39.7	1,377	39.1
Non-binary or self-described	5	0.6	5	0.6	2	0.2	3	0.3	15	0.4
**Age (years)**										
18–39	304	34.3	305	34.2	278	31.8	335	38.7	1,222	34.7
40–59	302	34.1	283	31.8	283	32.4	273	31.5	1,141	32.4
60+	281	31.7	303	34.0	313	35.8	258	29.8	1,155	32.8
**Race and ethnicity** ^1^										
NH White	643	73.2	654	73.8	634	73.2	621	72.3	2,552	73.1
Hispanic (any race)	83	9.4	94	10.6	85	9.8	94	10.9	356	10.2
NH Black or African American	80	9.1	87	9.8	76	8.8	89	10.4	332	9.5
NH Asian or Pacific Islander	33	3.8	29	3.3	38	4.4	31	3.6	131	3.8
NH Other/Multi-racial	40	4.6	22	2.5	33	3.8	24	2.8	119	3.4
**Education**										
High school diploma or less	292	33.2	311	35.1	291	33.6	294	34.2	1,188	34.1
Associate or technical degree	191	21.7	197	22.3	214	24.7	204	23.7	806	23.1
4-year college degree	269	30.6	250	28.2	250	28.9	255	29.7	1,024	29.3
Graduate degree	127	14.4	127	14.4	111	12.8	106	12.3	471	13.5
**Household income**										
Lower ($0 to <$35,000)	244	27.8	294	33.2	271	31.3	255	29.7	1,064	30.5
Middle ($35,000 to <$74,999)	321	36.6	319	36.0	316	36.5	333	38.8	1,289	37.0
Higher (≥$74,999)	313	35.6	272	30.7	278	32.1	271	31.5	1,134	32.5
**Red meat consumption**										
1 time/week	141	15.9	130	14.6	125	14.3	129	14.9	525	14.9
2–3 times/week	447	50.4	430	48.3	416	47.6	403	46.5	1,696	48.2
4–6 times/week	187	21.1	204	22.9	217	24.8	192	22.2	800	22.7
≥ 1 time/day	112	12.6	127	14.3	116	13.3	142	16.4	497	14.1
**Interest in health** ^2^										
Low	93	10.6	104	11.7	95	11.0	93	10.8	385	11.0
Moderate-low	233	26.5	231	26.1	252	29.1	231	26.9	947	27.1
Moderate-high	432	49.1	447	50.5	409	47.2	444	51.7	1,732	49.6
High	121	13.8	104	11.7	110	12.7	91	10.6	426	12.2
**Interest in sustainability** ^3^										
Low	69	7.9	70	7.9	72	8.3	68	7.9	279	8.0
Moderate-low	176	20.0	152	17.2	164	18.9	151	17.6	643	18.4
Moderate-high	362	41.2	382	43.1	385	44.5	396	46.1	1,525	43.7
High	271	30.9	282	31.8	245	28.3	244	28.4	1,042	29.9

^1^Self-reported identity. NH = Non-Hispanic; NH White and NH Black or African American exclude NH multi-racial; NH

Asian or Pacific Islander include NH Asian only, NH Pacific Islander only, and NH Asian and Pacific Islander

^2^Based on a scale of self-perceived dietary behavior [66]

^3^Based on the GREEN Scale [67]

### Effect of the red meat warning labels and tax on selection of food groups

As previously reported, the three interventions (red meat warning labels, red meat tax, and combined warning labels + tax) had the intended effects on overall selection of red meat (without consideration of processing or species), with participants in the intervention trial arms selecting 0.3 to 0.8 fewer items with red meat compared to participants in the control arm [49].

The three interventions affected selection of subcategories of red meat, as well as selection of other food groups (Table 2). For example, compared to the control group, participants exposed to the tax (-0.16, 95% CI: -0.27 -0.04, *q* = 0.014) or the combined warning labels + tax (-0.21, 95% CI: -0.33, -0.10, *q =* 0.001) selected fewer items containing unprocessed meat, driven by reductions in unprocessed beef. Additionally, all three interventions (warning labels, tax, or combined warning labels + tax) led participants to select significantly fewer items containing processed meat. These reductions were driven primarily by reductions in processed pork, with participants in the warning labels arm selecting 0.14 fewer items with processed pork (95% CI: -0.24, -0.05, *q* = 0.004), those in the tax arm selecting 0.23 fewer items with processed pork (95% CI: -0.33, -0.14, *q* < 0.001), and those in the combined warning labels + tax arm selecting 0.36 fewer items with processed pork than the control group (95% CI: -0.45, -0.27, *q* < 0.001). The combined warning labels + tax, but not the other two interventions, led participants to select more items containing processed poultry than the control (0.12, 95% CI: 0.06, 0.18, *q* = 0.001).

**Table 2:** Number of items selected from different food groups, by trial arm

	Control			Warning Labels				Tax				Combined Warning Labels + Tax			
	Mean	95% CI		Contrast	95% CI		*q* ^1^	Contrast	95% CI		*q* ^1^	Contrast	95% CI		*q* ^1^
Unprocessed meat	2.65	(2.57,	2.73)	-0.08	(-0.20,	0.03)	0.163	-0.16	(-0.27,	-0.04)	**0.014**	-0.21	(-0.33,	-0.10)	**0.001**
Beef	1.43	(1.35,	1.50)	-0.10	(-0.20,	0.00)	0.051	-0.18	(-0.28,	-0.08)	**0.001**	-0.32	(-0.42,	-0.22)	**< 0.001**
Pork	0.03	(0.02,	0.04)	0.00	(-0.02,	0.01)	0.931	-0.02	(-0.03,	0.00)	0.089	-0.01	(-0.02,	0.01)	0.931
Poultry	1.07	(1.01,	1.13)	0.00	(-0.09,	0.08)	0.913	0.03	(-0.05,	0.12)	0.913	0.10	(0.01,	0.19)	0.073
Fish & seafood	0.07	(0.05,	0.09)	-0.01	(-0.03,	0.02)	0.879	-0.02	(-0.04,	0.01)	0.606	-0.01	(-0.04,	0.02)	0.879
Other ruminant^2^	0.01	(0.00,	0.02)	0.00	(-0.02,	0.01)	> 0.999	0.00	(-0.02,	0.01)	> 0.999	-0.01	(-0.02,	0.01)	0.885
Processed meat	2.35	(2.28,	2.42)	-0.12	(-0.22,	-0.02)	**0.020**	-0.16	(-0.26,	-0.06)	**0.004**	-0.26	(-0.36,	-0.16)	**< 0.001**
Beef	0.08	(0.06,	0.11)	0.00	(-0.03,	0.02)	0.967	0.01	(-0.02,	0.05)	0.967	-0.02	(-0.05,	0.00)	0.317
Pork	1.82	(1.75,	1.88)	-0.14	(-0.24,	-0.05)	**0.004**	-0.23	(-0.33,	-0.14)	**< 0.001**	-0.36	(-0.45,	-0.27)	**< 0.001**
Poultry	0.44	(0.40,	0.48)	0.03	(-0.03,	0.09)	0.377	0.06	(0.00,	0.12)	0.113	0.12	(0.06,	0.18)	**0.001**
Fish & seafood	0.00	(0.00,	0.00)	0.00	(0.00,	0.00)	0.951	0.00	(0.00,	0.00)	0.951	0.00	(0.00,	0.00)	> 0.999
Vegetarian	3.57	(3.47,	3.67)	0.20	(0.05,	0.34)	**0.008**	0.32	(0.18,	0.47)	**< 0.001**	0.49	(0.35,	0.64)	**< 0.001**
Cheese	0.68	(0.62,	0.73)	0.20	(0.12,	0.28)	**< 0.001**	0.27	(0.18,	0.35)	**< 0.001**	0.33	(0.24,	0.41)	**< 0.001**
Other dairy	0.05	(0.03,	0.08)	-0.02	(-0.05,	0.01)	0.625	0.00	(-0.03,	0.04)	0.914	-0.02	(-0.05,	0.01)	0.625
Eggs	0.04	(0.02,	0.05)	0.00	(-0.02,	0.02)	> 0.999	0.00	(-0.02,	0.02)	> 0.999	0.02	(-0.01,	0.05)	0.434
Tofu	0.05	(0.04,	0.07)	0.01	(-0.02,	0.03)	> 0.999	0.00	(-0.02,	0.02)	> 0.999	0.01	(-0.01,	0.03)	> 0.999
Meat mimic^3^	0.19	(0.16,	0.22)	0.01	(-0.03,	0.05)	> 0.999	-0.01	(-0.05,	0.04)	> 0.999	0.03	(-0.02,	0.07)	0.796
Pulses^4^	0.23	(0.20,	0.26)	0.02	(-0.02,	0.06)	0.409	0.06	(0.01,	0.10)	**0.026**	0.05	(0.00,	0.09)	0.107
Nuts & seeds	0.23	(0.21,	0.26)	0.03	(-0.01,	0.07)	0.336	0.03	(-0.01,	0.07)	0.336	0.03	(-0.01,	0.07)	0.336
Grains & potatoes	1.95	(1.90,	1.99)	0.00	(-0.06,	0.07)	> 0.999	0.01	(-0.05,	0.08)	> 0.999	0.07	(0.00,	0.15)	0.123
Vegetables	0.14	(0.10,	0.18)	-0.06	(-0.10,	-0.01)	0.073	-0.04	(-0.09,	0.01)	0.259	-0.02	(-0.07,	0.04)	0.512

^1^Corrected for 3 comparisons (each trial arm compared to control) for each food group using a Bonferroni-Holm correction

^2^Lamb and bison

^3^Plant-based products that aim to simulate meat, for example, Beyond Beef, Impossible Beef, veggie sausage, or mock “chik’n” nuggets

^4^Mature seeds from the legume family: beans, lentils, or peas

Other differences between arms included that all three interventions led participants to select more vegetarian items overall and more items with cheese than the control arm. Additionally, the tax arm led participants to select more items with pulses than the control arm. By contrast, the three interventions did not affect selection of unprocessed pork, unprocessed poultry, processed beef, fish or seafood (processed or unprocessed), eggs, and dairy other than cheese. Likewise, except for pulses in the tax arm, the three interventions did not affect selection of plant-based items (pulses, nuts & seeds, tofu, meat mimics, grains & potatoes, and vegetables). Supplemental Table 6 shows the mean counts for arms, rather than contrasts.

### Effects by item type

Results showed both similarities and differences when stratifying by item type (i.e., when analyzing selections attributed to the pizza shopping list item, to the burrito shopping list item, etc., Supplemental Tables 7–12). For some item types, the results were similar to the overall results. For example, when analyzing burrito and burger patty selections, all three interventions led participants to select fewer items containing unprocessed beef (though for burger patties, these effects were significant only for the combined warning labels + tax arm), similar to the overall results. When examining pizza, sandwich and taco filling, and breakfast sausage selections, the combined warning labels + tax intervention led participants to select fewer items containing processed pork, as seen in the overall results. Also similar to the overall results, when examining breakfast sausage selections, the warning labels led participants to select fewer items containing processed pork and the combined warning labels + tax intervention led participants to select more items containing processed poultry. Likewise, when examining pizza and burritos selections, the combined warning labels + tax intervention led participants to select more items containing cheese, similar to the overall results.

For other item types, results differed from the overall results. For example, for analyses of burritos, the combined warning labels + tax intervention led participants to select more items containing unprocessed poultry, while no such effect was seen in the overall results. Additionally, for frozen meals, none of the interventions affected selections any food group compared to the control. Finally, for analyses of each item type, none of the interventions affected selections of items containing pulses, in contrast to the overall results which found that the tax intervention led participants to select more items containing pulses.

## Discussion

In this large randomized controlled trial, we found that participants exposed to warning labels or a tax on red meat selected fewer products containing processed red meat and (for taxes or warning labels + taxes) fewer products containing unprocessed red meat than participants not exposed to these policies. These reductions were driven by reductions in selection of processed pork and unprocessed beef. At the same time, the interventions affected selection of non-red-meat foods, including leading to higher selection of items containing cheese, pulses, and processed poultry, depending on the intervention. Together, these results indicate that implementing warning labels or taxes for red meat could have a range of effects on the types of foods consumers buy, beyond effects on red meat.

The observed pattern of substitution has five potential implications for population health. First, the reduction in selection of red meat could improve population health outcomes given that high consumption of red meat is associated with increased risk of coronary heart disease [52] and type 2 diabetes [48]. Second, the reduction in red meat selection overall was driven in part by reductions in processed red meat, and specifically by reductions in selection of processed pork; this reduction could be especially important for population health because high consumption of processed red meat is considered even more harmful to health than unprocessed red meat [53–55]. Third, participants exposed to only the tax selected more items containing pulses. If this increase is translated into increases in pulse consumption, it could improve certain health outcomes, given that replacing red meat with pulses has been associated with favorable changes in cholesterol [56] and reduced risk of coronary heart disease [42] and type 2 diabetes [57]. Fourth, participants exposed to the warning labels or tax also selected more items containing cheese. This change could have mixed effects on health: cheese tends to be high in saturated fat and sodium, nutrients that most Americans overconsume and that contribute to diet-related chronic diseases [58, 59]. However, some prospective cohort studies suggest that consuming cheese in place of red and processed meat—the substitution implied by our results—can reduce risk of heart disease and stroke [60, 61]. Fifth, participants exposed to both the warning labels and the tax simultaneously also selected more items containing processed poultry. This increase could offset some of the potential health benefits of the interventions, given the documented health harms of consuming processed meat (which is often defined to include processed poultry [62, 63]). Future studies should identify strategies for encouraging consumers to replace red meat with substitutes that are typically healthier than processed poultry, such as pulses or unprocessed poultry.

The observed pattern of substitution also has three potential implications for environmental sustainability. First, the reduction in selection of red meat could attenuate several environmental harms from food production, given that red meat is a major contributor to greenhouse gas emissions [15–19], air and water pollution [7, 15], deforestation [20, 21], and biodiversity loss [2, 22]. Second, the reduction in red meat selection overall was driven in part by a reduction in unprocessed beef; this reduction is especially important for environmental sustainability because compared to other animal-source foods, beef production generates a disproportionate amount of greenhouse gas emissions per kilogram of food (e.g., as much as 10 times the greenhouse gas emissions of poultry and 8 times that of pork [15]). Third, the warning labels and tax led participants to shift toward purchasing items containing poultry (specifically processed poultry) and items containing cheese. Although less greenhouse-gas-intensive than beef, producing poultry and cheese generates more greenhouse gas emissions than producing other potential substitutes for red meat like pulses, and Americans already consume more poultry and dairy products than recommended by the EAT-Lancet reference diet [64]. To maximize the environmental benefits of red meat warning labels and taxes, policymakers could explore coupling these policies with educational campaigns to promote substitutions to plant-based foods rather than animal products.

The pattern of substitution varied across the specific item types we asked participants to select in the trial (i.e., across the shopping list categories). This variation may have been driven by differences in what substitutes were available and perceived as acceptable in each category. For example, our results might suggest that consumers view cheese pizza as an acceptable substitute for pepperoni pizza (hence the interventions led participants to select more items with cheese in the pizza category) but do not view sliced cheese as an acceptable substitute for red-and-processed meat sandwich and taco fillings like roast beef or ground beef (hence the interventions did not lead participants to select more items with cheese in this category). The variation in substitution results by item type might also reflect differences in common ingredients in each of the item types. For example, the interventions did not lead to a reduction in unprocessed meat from pizza selections, perhaps because the meat on pizza is typically processed (e.g., pepperoni, sausage, ham), so the interventions were unlikely to reduce selection of unprocessed meats for this item type. As another example, we observed a reduction in processed meat when examining selections overall, but not when examining selections of burger patties, perhaps because most burger patties were, by our definition, unprocessed. The variation across item types implies that the effect of red meat warning labels or taxes could vary based on context (e.g., whether people are shopping for ingredients versus mixed or prepared dishes) and highlights the importance of capturing whole-diet impacts of policies focused on red meat, rather than examining only some food groups or types of selections.

Our results align with the small number of studies that have used simulation modeling or randomized experiments to examine the impact of similar interventions on selection of non-red-meat foods. One simulation modeling study, for example, projected that a tax on both red and processed meat would increase purchases of poultry, dairy, and eggs, in line with our findings that the red meat tax increased selection of some types of poultry [33]. Similarly, one randomized trial of climate warning labels on restaurant menu items containing red meat found that the warnings increased participants’ likelihood of selecting items with chicken or fish and their likelihood of selecting salads [39], and another found that health messages about red meat increased likelihood of selecting a restaurant menu item containing poultry or fish and environmental messages increased likelihood of selecting a vegetarian item [65]. Together with prior literature, our results highlight the importance of examining consumers’ overall purchase patterns in response to policies targeting red meat, including purchases of both red meat and non-red-meat foods.

Strengths of this study include the large, diverse sample, the randomized controlled design, the use of a naturalistic online grocery store that closely mimicked the experience of shopping in a real online grocery store, and the disaggregation of meat processing and species (i.e., type of animal the meat comes from) that is missing from many studies [62]. We also note six limitations. First, although we incentivized participants to select items they actually wished to receive, participants’ selections were hypothetical choices made in the context of a naturalistic online grocery store. Second, we asked participants to select specific types of food using a shopping list. Although the items on the shopping list included both ingredients and entire meals, and represented popular foods (e.g., pizzas, burritos), our results may not generalize to settings in which consumers are not choosing these types of food, or to consumer food choices outside the US context. Third, we categorized foods into food groups based on the presence of key ingredients (such as red and processed meat) but were unable to examine the amount of these ingredients in each food. Fourth, we applied the warning labels and tax to products with any red meat, even small amounts, and this might not represent how these policies would be implemented in the real world. A real tax might scale with the amount of red meat in a product and therefore produce different responses for the purchases of ingredients versus entrees (e.g., the relative price increase of ground beef would be much higher than that of a pizza containing pepperoni). Fifth, the study was powered to detect a difference (Cohen’s d = 0.13) in red meat selections between intervention arms and the control, and we may have lacked power to detect meaningful differences in the smaller, more disaggregated food groups in this secondary analysis. Sixth, we examined the effects of displaying health and environmental warning labels simultaneously and so cannot estimate the effects of exposure to just health or just environmental warning labels. A previous US study found that health messages alone were more effective than environmental messages alone, but that a combined label with health and environmental messages was most effective [27]. Likewise, we examined only one tax rate. We selected a 30% tax rate based on prior research estimating optimal tax rates for red meat in the US; a lower tax rate would be expected to have a more modest impact on purchases.

## Conclusions

In this large randomized controlled trial, warning labels and a tax on red meat led to lower selection of items containing processed pork and unprocessed beef and higher selection of items containing processed poultry, cheese, and pulses. These changes could lead to some health and environmental benefits, though additional interventions are likely needed to achieve alignment with dietary patterns that would maximize human health and environmental sustainability.

### Electronic supplementary material

Below is the link to the electronic supplementary material.

Supplementary Material 1

## Data Availability

Data files are available from the Harvard Dataverse at https://doi.org/10.7910/DVN/OB8ZFJ.
