# Supplementary material for: Effects of red meat taxes and warning labels on food groups selected in a randomized controlled trial
Source: Int J Behav Nutr Phys Act. 2024 Apr 15;21:39. doi: 10.1186/s12966-024-01584-9 (PMC11020801; doi:10.1186/s12966-024-01584-9)
Supplement: Supplementary file 1 — Supplementary Material 1 [file 12966_2024_1584_MOESM1_ESM.docx]

**Supplemental Materials**

Effects of red meat taxes and warning labels on food groups selected in a randomized controlled trial

By Amelia Willits-Smith, Lindsey Smith Taillie, Lindsay M. Jaacks, Sarah M. Frank, and Anna H. Grummon

Contents

[Supplemental Table 1. Food ingredient code definitions 2](#_Toc162013254)

[Supplemental Methods 4](#_Toc162013255)

[Supplemental Figure 1. Assignment of mutually exclusive food groups 6](#_Toc162013256)

[Supplemental Table 2. Shopping list item definitions 7](#_Toc162013257)

[Supplemental Table 3. Frequency of food groups among items selected by participants 9](#_Toc162013258)

[Supplemental Table 4. Shopping list compliance 10](#_Toc162013259)

[Supplemental Table 5. Proportion of participants purchasing item types by trial arm 11](#_Toc162013260)

[Supplemental Table 6. Count of items selected from different food groups, by trial arm 12](#_Toc162013261)

[Supplemental Table 7. Count of items selected from different food groups, by shopping list task and trial arm: Pizza (N=3,046) 13](#_Toc162013262)

[Supplemental Table 8. Count of items selected from different food groups, by shopping list task and trial arm: Burrito (N=2,614) 14](#_Toc162013263)

[Supplemental Table 9. Count of items selected from different food groups, by shopping list task and trial arm: Burger patties (N=3,045) 15](#_Toc162013264)

[Supplemental Table 10. Count of items selected from different food groups, by shopping list task and trial arm: Breakfast sausage (N=2,800) 16](#_Toc162013265)

[Supplemental Table 11. Count of items selected from different food groups, by shopping list task and trial arm: Frozen meal (N=2,769) 17](#_Toc162013266)

[Supplemental Table 12. Count of items selected from different food groups, by shopping list task and trial arm: Sandwich and taco fillings (N=3,364) 18](#_Toc162013267)

## Supplemental Table 1. Food ingredient code definitions

| **Code** | **Includes** |
| --- | --- |
| Processed meat^1^ | Flesh foods that have been transformed through salting, curing, fermentation, smoking, or other processes for preservation or flavor enhancement.  Bacon  Sausage  Hot dogs  Deli/lunch meat  Philly cheesesteak  Imitation crab  Pulled chicken, pork, or beef if smoked |
| Unprocessed meat | Flesh foods not transformed as above. |
| Beef | Meat from cattle. |
| Other ruminant | Sheep, lamb, goat, bison, buffalo, venison, elk. |
| Pork | Meat from pigs. |
| Poultry | Chicken, turkey, duck, quail. |
| Fish & seafood | Fish, shellfish, bivalves. |
| Vegetarian | Does not include meat (flesh food). |
| Cheese | Cow or goat cheese. Includes cream cheese, cottage cheese. |
| Other dairy | Milk, yogurt, sour cream when they are a standalone ingredient.  Excludes:  Soy or plant-based milks, yogurts, etc.  Milk as a sub-ingredient (e.g. in a dressing that comes with a salad, or the milk that makes up cheese)  Powdered, extracts, or other highly processed components used in small amounts, e.g. sour cream powder seasoning the tortilla chips in a salad, or whey used in a seasoning. |
| Eggs | Includes liquid egg whites.  Excludes:  Eggs as a sub-ingredient, e.g. egg yolk in Caesar dressing that comes with a salad  Egg white or dried egg white in last few ingredients – binder only, not a common sense “egg” food |
| Classic meat alternative^2^ | Tofu  Excludes:  Highly processed soy products (e.g. textured soy protein, soy protein isolate)  Products described like they are meat (see below) |
| Meat mimicry product | Beyond Beef  Impossible Burger  Description contains a word usually applied to meats (e.g. “sausage”), but product is meatless |
| Pulses | All beans and lentils, including soybeans if they are whole.  Excludes:  Processed soy products  Processed pea or bean protein |
| Nuts/seeds | All nuts, seeds, and butters made from them. Includes variations like reduced fat peanut butter.  Excludes:  Nut-based milks |

Supplemental Table 1 (continued)

| **Code** | **Includes** |
| --- | --- |
| Mixed meat product | If the product has more than one type of meat in it, code this as 1/yes, and complete the next three items based on the order the meats appear in ingredients list |
| Primary meat  Secondary meat  Tertiary meat | 1=Beef  2=Pork  3=Poultry  4=Other ruminant  5=Fish & seafood |
| Grains & potatoes | All items that count toward bread loaf and tortilla shopping list item, as well as:  Boxed mashed potatoes  Potato chips  Popcorn, crackers  Pasta without sauce |
| Vegetable | Includes: Onion rings  Excludes: Herbs |
| Beverage (not included in analysis) | Water, juice, smoothies  Coffee beans/grounds  Tea bags  Fruit drink mixes  Coffee creamer  Frozen juice concentrate |
| Dessert (not included in analysis) | Breakfast Muffins shelf^3^  Toaster pastries  Cinnamon rolls |
| Dairy alternative (not included in analysis) | Soy or other plant-based milk, yogurt, creamers, cheese, etc. |
| Fats (not included in analysis) | Oils, butter, margarine spreads |
| Condiments (not included in analysis) | Jam  Hot sauce, taco sauce  Ketchup  Mustard  Steak sauce  Sweet & sour sauce |
| Other (not included in analysis) | Herbs, spices  Corn starch  Breading mix  Stevia  Baby foods, infant formula  Broth |

^1^Definition from Bouvard, V., Loomis, D., Guyton, K. Z., Grosse, Y., Ghissassi, F. el, Benbrahim-Tallaa, L., et al. (2015). Carcinogenicity of consumption of red and processed meat. The Lancet Oncology, 16, 1599–1600. <https://doi.org/10.1016/S1470-2045(15)00444-1>. Note: processed food *products* may have meat that we did not classify as processed meat. For example, a beef, bean, and cheese chimichangas: “beef” is one ingredient and we would code that as unprocessed, but other elements like dough conditioners, etc. indicate this a processed food overall.

^2^This group theoretically included tempeh and seitan, but those did not show up in participant purchases in the trial.

^3^The data scraped from a major US food retailer included “departments” (e.g., “Eggs & Dairy”) and more specific “shelves” (e.g., “Frozen International Appetizers,” “Packaged Sliced Meat,” “Hamburger Buns,” or “Block Cheese”) that were used to build navigation in the simulated online grocery store. Food products could appear in more than one shelf.

## Supplemental Methods

***Details on assignment of food group***

We began grouping by first evaluating whether the product contained any meat, poultry, fish, or seafood. If it did, it was grouped by the species or species type, and whether that meat was processed or unprocessed. So, for example, a cheeseburger would be categorized as unprocessed beef. When more than one type of meat was present in an item, the first one to appear in the ingredients list was used to categorize the food (e.g., a bacon cheeseburger would also be categorized as unprocessed beef), since the order of ingredients reflects relative amounts of each ingredient. If the product did not contain any meat, poultry, fish, or seafood, but did contain cheese, it was categorized as cheese. This process continued in the following order, prioritizing the presence of animal products and roughly in the order of an ingredient’s environmental impact during production: (1) Meat, poultry, fish or seafood; (2) cheese; (3) other dairy; (4) eggs; (5) tofu; (6) meat mimics (plant-based products described as “sausage,” “beef,” “chik’n,” etc.); (7) pulses (mature seeds from the legume family: beans, lentils, or peas); (8) nuts or seeds (including peanuts); (9) grains or potatoes; and (10) vegetables. This process is illustrated in **Supplemental Figure 1**.

Because nutrition facts information for packaged products does not include information on quantity of ingredients (e.g., grams of beef in a cheeseburger), the presence of even a small amount of a target ingredient in a food was coded and used to assign food category. Exceptions to this included bread, tortillas, and some items that were completely off the shopping list. “Loaf of bread” and “pack of tortillas” were two items from the shopping list (see **Supplemental Table 2**). So even if some versions of these contained small amounts of milk, butter, or lard, they were still categorized as grains & potatoes. While the shopping list was intended to elicit purchases of foods that are the dominant sources of Americans’ red and processed meat intake—a list with only savory items—participants selected a range of dessert products such as muffins, donuts, pie, candy, and ice cream. Since these off-list items would not be expected to differ across trial arms, and are unlikely substitutes for red meat, they were combined into a single group rather than separating them by dairy or grain content. Similarly, beverages containing small amounts of milk (e.g., hot cocoa mix) were categorized as beverages, and oils, margarine, and butter were categorized as fats. Condiments were combined into a single group even if they contained some eggs (e.g, mayonnaise) or dairy (e.g. ranch dressing).

Our ability to accurately code and group each food product was also dependent on the accuracy of the online data scraping that was used to build the simulated grocery store. For items with implausible or missing ingredients (less than 10 items), we used information from the manufacturer’s website. We also used manufacturer’s websites (e.g., package images or text food descriptions) if the food description or ingredients in our dataset was not sufficient to distinguish processed versus unprocessed meat (e.g., to find whether pulled pork was smoked).

## Supplemental Figure 1. Assignment of mutually exclusive food groups

**
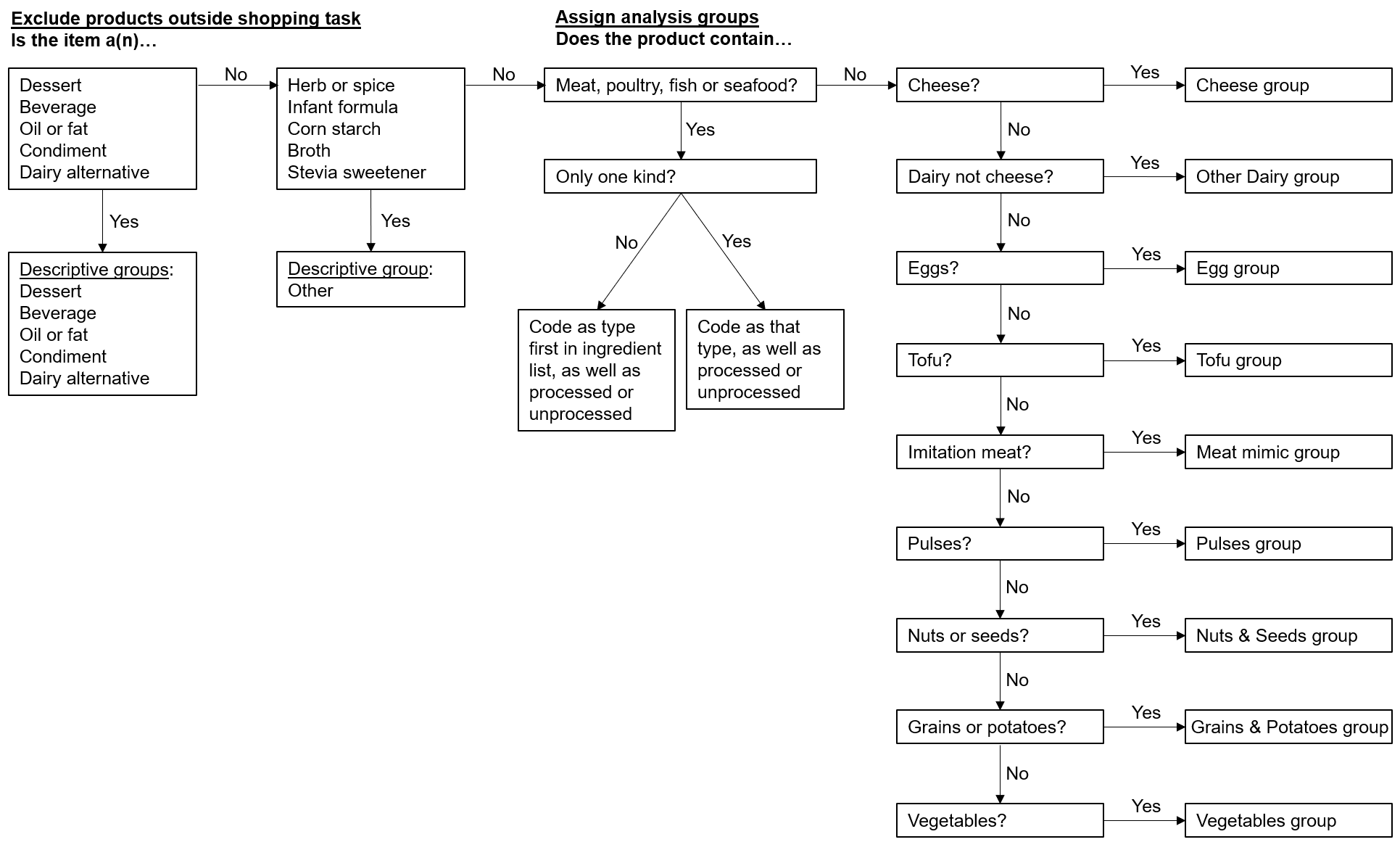
**

## Supplemental Table 2. Shopping list item definitions

| **Shopping list item** | **Includes** | **Excludes** |
| --- | --- | --- |
| Loaf of bread | Cinnamon raisin bread  Rolls/buns  Bagels  Flatbread, naan, pita  Breadsticks  Biscuits, crescent rolls, croissants  Brioche bread  Fresh or frozen | Muffins, cakes (==off list)  Tortillas (==tortillas) |
| Pack of tortillas | Low carb versions described as wraps  Fresh or frozen | Flatbread & naan (==bread) |
| Pizza | Fresh or frozen  Lunchables if “pizza” is in the description  Bagel bites if “pizza” is in description  Foods on “Pizza essentials” shelf^1^, which includes some of the pepperoni entries  Pepperoni |  |
| Burrito | All Mexican mixed dishes, fresh or frozen (e.g. taqauitos, chimichangas) |  |
| Burger patties | All ground meats, whether bulk or “patty” form, and fresh or frozen  Chicken patties  Veggie patties of all types (description includes “burger” or “patties”; includes “chik’n”) |  |
| Breakfast sausages | Items on Breakfast Sausage shelf^1^ (even if they are also on Dinner Sausage shelf^1^)  Italian sausages (even if not on Breakfast Sausage shelf^1^)  Veggie sausages | Sausages on Dinner Sausage shelf^1^ only (==sandwich)  Chorizo (==taco) |
| Frozen individual meal | Frozen mixed foods, meals, or meal starters  Uncrustables  Loaded baked potato skins  Filled soft pretzels  Breaded/fried chicken products (nuggets, tenders, strips)  Chicken wings (if frozen)  Chicken nuggets or tenders  Mock “chik’n” veggie tenders  Meatloaf  French fries | Steak, whole chicken, poultry parts other than wings (==sandwich)  Ground poultry or beef (==patties)  Mexican foods of any type (==burrito)  Pure starch/side dish, e.g. soft pretzels, hush puppies, onion rings (==off list) |
| Sandwich filling  (combined with taco in Stata) | Bacon  Salami  Almost all cheese (block, shredded, sliced, cream, cottage)  Items on Dinner Sausage shelf^1^ but not Breakfast Sausage shelf^1^ (includes hot dogs)  Chicken parts  Beef or pork if not ground, or does not have “patty” in product name, e.g. beef steak  Pork ribs  Meatloaf  Condiments and spreads: mayonnaise, ketchup, mustard, steak sauce  Hummus | String cheese (==off list)  Ground meats (==patties)  Chicken or mock “chik’n” patties (==patties)  Chicken wings (==frozen) |

Supplemental Table 2 (continued)

| **Shopping list item** | **Includes** | **Excludes** |
| --- | --- | --- |
| Taco filling  (combined with sandwich filling) | Chorizo  Most fresh or frozen potatoes  Vegetables, all forms (incl fresh garlic, ginger)  Plantain  Avocado, guacamole  Beans (including pork & beans, ham & beans)  Chili (with or without beans)  Rice, quinoa  Dips, including salsa  Sour cream, Mexican crema  Plain yogurt  Chipotle Black Bean veggie crumbles | Hash brown patties, french fries (==frozen)  Mashed potatoes (==off list) |
| Not a shopping list item | Flavored yogurt  Cookies, cakes, etc.  Fruit  Packaged salads  Oils, butter, margarine  Herbs, spices, seasoning mixes  Other dry goods (e.g. pasta)  Pasta sauce  Shelf stable dinners/meals (e.g., mac & cheese, ramen)  Frozen starch/sides, e.g. soft pretzels, hush puppies, waffles  Mashed or scalloped potatoes (refrigerated or instant mixes)  Beverages  Milk, coffee creamer  Chips, crackers, popcorn  Baby food, infant formula |  |

^1^The data scraped from a major US food retailer included “departments” (e.g., “Eggs & Dairy”) and more specific “shelves” (e.g., “Frozen International Appetizers,” “Packaged Sliced Meat,” “Hamburger Buns,” or “Block Cheese”) that were used to build navigation in the simulated online grocery store. Food products could appear in more than one shelf.

## Supplemental Table 3. Frequency of food groups among items selected by participants

| **Food Group** | **N** | **%** |
| --- | --- | --- |
| **Analysis categories** |  |  |
| Unprocessed meat |  |  |
| Beef | 144 | 5.3 |
| Pork | 24 | 0.9 |
| Poultry | 224 | 8.3 |
| Fish & seafood | 60 | 2.2 |
| Other Ruminant^1^ | 2 | 0.1 |
| Processed meat |  |  |
| Beef | 52 | 1.9 |
| Pork | 273 | 10.1 |
| Poultry | 135 | 5.0 |
| Fish & seafood | 2 | 0.1 |
|  |  |  |
| Vegetarian |  |  |
| Cheese | 215 | 8.0 |
| Other dairy | 66 | 2.4 |
| Eggs | 33 | 1.2 |
| Tofu | 4 | 0.1 |
| Meat Mimic | 27 | 1.0 |
| Pulses^2^ | 68 | 2.5 |
| Nuts & Seeds | 89 | 3.3 |
| Grains & Potatoes | 409 | 15.1 |
| Vegetables | 179 | 6.6 |
|  |  |  |
| **Excluded/informational categories** |  |  |
| Beverages | 277 | 10.2 |
| Dessert | 178 | 6.6 |
| Fruit | 89 | 3.3 |
| Fats | 40 | 1.5 |
| Condiments | 35 | 1.3 |
| Dairy alternative | 14 | 0.5 |
| Other | 64 | 2.4 |
| Total | 2703 | 100.0 |

^1^Lamb and bison.

^2^Plant-based products that aim to simulate meat, for example, Beyond Beef, Impossible Beef, veggie sausage, or mock “chik’n” nuggets.

^3^Mature seeds from the legume family: beans, lentils, or peas.

## Supplemental Table 4. Shopping list compliance

| **Shopping list compliance (max=8)** | **Trial Arm** | | | | | | | | **Total** | |
| --- | --- | --- | --- | --- | --- | --- | --- | --- | --- | --- |
|  | **Control** | | **Warning Labels** | | **Tax** | | **Combined Warning Labels + Tax** | |  |  |
|  | **N** | **Row %** | **N** | **Row %** | **N** | **Row %** | **N** | **Row %** | **N** | **Overall %** |
| 0 | 11 | 33.3 | 11 | 33.3 | 4 | 12.1 | 7 | 21.2 | 33 | 0.94 |
| 1 | 25 | 25.8 | 25 | 25.8 | 25 | 25.8 | 22 | 22.7 | 97 | 2.76 |
| 2 | 46 | 24.6 | 49 | 26.2 | 46 | 24.6 | 46 | 24.6 | 187 | 5.32 |
| 3 | 32 | 25.6 | 26 | 20.8 | 38 | 30.4 | 29 | 23.2 | 125 | 3.55 |
| 4 | 15 | 22.7 | 21 | 31.8 | 15 | 22.7 | 15 | 22.7 | 66 | 1.88 |
| 5 | 17 | 24.3 | 12 | 17.1 | 17 | 24.3 | 24 | 34.3 | 70 | 1.99 |
| 6 | 36 | 21.3 | 45 | 26.6 | 42 | 24.9 | 46 | 27.2 | 169 | 4.80 |
| 7 | 171 | 22.0 | 204 | 26.2 | 191 | 24.6 | 212 | 27.2 | 778 | 22.11 |
| 8 | 534 | 26.8 | 498 | 25.0 | 496 | 24.9 | 465 | 23.3 | 1,993 | 56.65 |
| Total | 887 |  | 891 |  | 874 |  | 866 |  | 3,518 |  |

## Supplemental Table 5. Proportion of participants purchasing item types by trial arm

| **Item Type** | **Arm** | | | | | | | | **Total** | |
| --- | --- | --- | --- | --- | --- | --- | --- | --- | --- | --- |
|  | **Control** | | **Warning Labels** | | **Tax** | | **Combined Warning Labels + Tax** | |  |  |
|  | **N** | **%** | **N** | **%** | **N** | **%** | **N** | **%** | **N** | **%** |
| Pizza | 768 | 86.6 | 772 | 86.6 | 755 | 86.4 | 751 | 86.7 | 3,046 | 86.6 |
| Burrito | 683 | 77.0 | 653 | 73.3 | 640 | 73.2 | 638 | 73.7 | 2,614 | 74.3 |
| Burger patties | 769 | 86.7 | 783 | 87.9 | 756 | 86.5 | 737 | 85.1 | 3,045 | 86.6 |
| Breakfast sausage | 707 | 79.7 | 701 | 78.7 | 709 | 81.1 | 683 | 78.9 | 2,800 | 79.6 |
| Frozen meal | 706 | 79.6 | 697 | 78.2 | 683 | 78.1 | 683 | 78.9 | 2,769 | 78.7 |
| Bread | 819 | 92.3 | 816 | 91.6 | 816 | 93.4 | 807 | 93.2 | 3,258 | 92.6 |
| Tortillas | 747 | 84.2 | 754 | 84.6 | 729 | 83.4 | 738 | 85.2 | 2,968 | 84.4 |
| Sandwich or taco filling | 844 | 95.2 | 851 | 95.5 | 845 | 96.7 | 824 | 95.2 | 3,364 | 95.6 |

## Supplemental Table 6. Count of items selected from different food groups, by trial arm

| **Food Group** | **Control** | | | **Warning Labels** | | | **Tax** | | | **Combined Warning Labels+ Tax** | | |
| --- | --- | --- | --- | --- | --- | --- | --- | --- | --- | --- | --- | --- |
|  | **Mean** | **95% CI** | | **Mean** | **95% CI** | | **Mean** | **95% CI** | | **Mean** | **95% CI** | |
| Unprocessed meat | 2.65 | (2.57, | 2.73) | 2.57 | (2.48, | 2.65) | 2.49 | (2.41, | 2.57) | 2.44 | (2.36, | 2.52) |
| Beef | 1.43 | (1.35, | 1.50) | 1.33 | (1.26, | 1.39) | 1.24 | (1.17, | 1.31) | 1.11 | (1.04, | 1.17) |
| Pork | 0.03 | (0.02, | 0.04) | 0.03 | (0.02, | 0.04) | 0.02 | (0.01, | 0.02) | 0.03 | (0.01, | 0.04) |
| Poultry | 1.07 | (1.01, | 1.13) | 1.07 | (1.01, | 1.13 | 1.10 | (1.05 | 1.16) | 1.17 | (1.11, | 1.24) |
| Fish & seafood | 0.07 | (0.05, | 0.09) | 0.06 | (0.04, | 0.08) | 0.05 | (0.03, | 0.07) | 0.06 | (0.04, | 0.07) |
| Other ruminant^1^ | 0.01 | (0.00, | 0.02) | 0.01 | (0.00, | 0.02) | 0.01 | (0.00, | 0.01) | 0.00 | (0.00, | 0.01) |
|  |  |  |  |  |  |  |  |  |  |  |  |  |
| Processed meat | 2.35 | (2.28, | 2.42) | 2.23 | (2.16, | 2.30) | 2.19 | (2.12, | 2.26) | 2.09 | (2.02, | 2.16) |
| Beef | 0.08 | (0.06, | 0.11) | 0.08 | (0.06, | 0.10) | 0.10 | (0.07, | 0.13) | 0.06 | (0.05, | 0.08) |
| Pork | 1.82 | (1.75, | 1.88) | 1.67 | (1.60, | 1.74) | 1.58 | (1.52, | 1.65) | 1.46 | (1.39, | 1.52) |
| Poultry | 0.44 | (0.40, | 0.48) | 0.47 | (0.43, | 0.51) | 0.50 | (0.46, | 0.54) | 0.56 | (0.52, | 0.61) |
| Fish & seafood | 0.00 | (0.00, | 0.00) | 0.00 | (0.00, | 0.00) | 0.00 | (0.00, | 0.00) | 0.00 | (0.00, | 0.00) |
|  |  |  |  |  |  |  |  |  |  |  |  |  |
| Vegetarian | 3.57 | (3.47, | 3.67) | 3.76 | (3.66, | 3.87) | 3.89 | (3.78, | 3.99) | 4.06 | (3.95, | 4.17) |
| Cheese | 0.68 | (0.62, | 0.73) | 0.88 | (0.82, | 0.94) | 0.94 | (0.88, | 1.00) | 1.00 | (0.94, | 1.06) |
| Other dairy | 0.05 | (0.03, | 0.08) | 0.03 | (0.02, | 0.05) | 0.06 | (0.03, | 0.08) | 0.03 | (0.02, | 0.05) |
| Eggs | 0.04 | (0.02, | 0.05) | 0.04 | (0.02, | 0.05) | 0.04 | (0.03, | 0.06) | 0.06 | (0.03, | 0.09) |
| Tofu | 0.05 | (0.04, | 0.07) | 0.06 | (0.04, | 0.07) | 0.05 | (0.03, | 0.06) | 0.06 | (0.04, | 0.08) |
| Meat mimic^2^ | 0.19 | (0.16, | 0.22) | 0.20 | (0.17, | 0.23) | 0.19 | (0.16, | 0.22) | 0.22 | (0.19, | 0.25) |
| Pulses^3^ | 0.23 | (0.20, | 0.26) | 0.24 | (0.21, | 0.28) | 0.29 | (0.25, | 0.32) | 0.27 | (0.24, | 0.31) |
| Nuts & seeds | 0.23 | (0.21, | 0.26) | 0.26 | (0.23, | 0.29) | 0.26 | (0.23, | 0.29) | 0.27 | (0.24, | 0.30) |
| Grains & potatoes | 1.95 | (1.90, | 1.99) | 1.95 | (1.91, | 2.00) | 1.96 | (1.91, | 2.01) | 2.02 | (1.97, | 2.08) |
| Vegetables | 0.14 | (0.10, | 0.18) | 0.08 | (0.05, | 0.11) | 0.10 | (0.07, | 0.13) | 0.12 | (0.08, | 0.16) |
|  |  |  |  |  |  |  |  |  |  |  |  |  |
| Excluded/informational |  |  |  |  |  |  |  |  |  |  |  |  |
| Beverages | 0.18 | (0.12, | 0.24) | 0.21 | (0.15, | 0.27) | 0.19 | (0.14, | 0.24) | 0.17 | (0.12, | 0.22) |
| Fruit | 0.10 | (0.07, | 0.13) | 0.09 | (0.06, | 0.12) | 0.10 | (0.07, | 0.13) | 0.08 | (0.05, | 0.10) |
| Desserts | 0.08 | (0.05, | 0.12) | 0.10 | (0.07, | 0.13) | 0.09 | (0.06, | 0.13) | 0.10 | (0.07, | 0.13) |
| Fats & oils | 0.03 | (0.02, | 0.05) | 0.04 | (0.02, | 0.05) | 0.04 | (0.02, | 0.06) | 0.04 | (0.02, | 0.06) |
| Condiments | 0.02 | (0.01, | 0.02) | 0.02 | (0.01, | 0.03) | 0.03 | (0.02, | 0.04) | 0.02 | (0.01, | 0.03) |
| Dairy alternatives | 0.01 | (0.00, | 0.02) | 0.01 | (0.00, | 0.02) | 0.00 | (0.00, | 0.01) | 0.01 | (0.00, | 0.01) |
| Other | 0.06 | (0.03, | 0.08) | 0.05 | (0.03, | 0.08) | 0.08 | (0.04, | 0.12) | 0.05 | (0.02, | 0.07) |

^1^Lamb and bison.

^2^Plant-based products that aim to simulate meat, for example, Beyond Beef, Impossible Beef, veggie sausage, or mock “chik’n” nuggets.

^3^Mature seeds from the legume family: beans, lentils, or peas.

## Supplemental Table 7. Count of items selected from different food groups, by shopping list task and trial arm: Pizza (N=3,046)

| **Food Group** | **Control**  **(n=768)** | | | **Warning Labels**  **(n=772)** | | | | **Tax**  **(n=755)** | | | | **Combined Warning Labels + Tax**  **(n=751)** | | | |
| --- | --- | --- | --- | --- | --- | --- | --- | --- | --- | --- | --- | --- | --- | --- | --- |
|  | Mean | 95% CI | | Contrast | 95% CI | | *q*^1^ | Contrast | 95% CI | | *q*^1^ | Contrast | 95% CI | | *q*^1^ |
| Unprocessed meat | 0.03 | (0.01, | 0.04) | 0.01 | (-0.01, | 0.03) | 0.778 | 0.00 | (-0.01, | 0.02) | 0.778 | -0.01 | (-0.02, | 0.00) | 0.552 |
| Beef | 0.01 | (0.00, | 0.02) | 0.00 | (-0.01, | 0.00) | 0.723 | 0.00 | (-0.01, | 0.01) | 0.978 | -0.01 | (-0.01, | 0.00) | 0.291 |
| Pork | -- |  |  | -- |  |  |  | -- |  |  |  | -- |  |  |  |
| Poultry | 0.02 | (0.01, | 0.03) | 0.01 | (0.00, | 0.03) | 0.414 | 0.00 | (-0.01, | 0.02) | >0.999 | 0.00 | (-0.02, | 0.01) | >0.999 |
| Fish & seafood | -- |  |  | -- |  |  |  | -- |  |  |  | -- |  |  |  |
| Other ruminant^2^ | -- |  |  | -- |  |  |  | -- |  |  |  | -- |  |  |  |
|  |  |  |  |  |  |  |  |  |  |  |  |  |  |  |  |
| Processed meat | 0.83 | (0.80, | 0.86) | -0.11 | (-0.15, | -0.06) | **<0.001** | -0.17 | (-0.21, | -0.12) | **<0.001** | -0.21 | (-0.26, | -0.16) | **<0.001** |
| Beef | × |  |  | × |  |  |  | × |  |  |  | × |  |  |  |
| Pork | 0.80 | (0.76, | 0.83) | -0.10 | (-0.15, | -0.05) | **<0.001** | -0.17 | (-0.22, | -0.12) | **<0.001** | -0.20 | (-0.26, | -0.15) | **<0.001** |
| Poultry | 0.03 | (0.02, | 0.04) | -0.01 | (-0.03, | 0.01) | 0.583 | 0.00 | (-0.02, | 0.02) | 0.929 | -0.01 | (-0.03, | 0.01) | 0.583 |
| Fish & seafood | -- |  |  | -- |  |  |  | -- |  |  |  | -- |  |  |  |
|  |  |  |  |  |  |  |  |  |  |  |  |  |  |  |  |
| Vegetarian | 0.20 | (0.17, | 0.23) | 0.08 | (0.03, | 0.13) | **0.001** | 0.15 | (0.10, | 0.19) | **<0.001** | 0.22 | (0.17, | 0.27) | **<0.001** |
| Cheese | 0.18 | (0.15, | 0.21) | 0.09 | (0.04, | 0.13) | **<0.001** | 0.16 | (0.12, | 0.21) | **<0.001** | 0.22 | (0.18, | 0.27) | **<0.001** |
| Other dairy | -- |  |  | -- |  |  |  | -- |  |  |  | -- | -- |  |  |
| Eggs | -- |  |  | -- |  |  |  | -- |  |  |  | -- | -- |  |  |
| Tofu | -- |  |  | -- |  |  |  | -- |  |  |  | -- | -- |  |  |
| Meat mimic^3^ | -- |  |  | -- |  |  |  | -- |  |  |  | -- | -- |  |  |
| Pulses^4^ | -- |  |  | -- |  |  |  | -- |  |  |  | -- | -- |  |  |
| Nuts/seeds | -- |  |  | -- |  |  |  | -- |  |  |  | -- | -- |  |  |
| Grains & potatoes | 0.01 | (0.00, | 0.02) | 0.00 | (-0.01, | 0.01) | 0.686 | -0.01 | (-0.02, | 0.00) | 0.059 | 0.01 | (-0.01, | 0.02) | 0.686 |
| Vegetables | 0.01 | (0.00, | 0.02) | -0.01 | (-0.02, | 0.00) | 0.402 | -0.01 | (-0.02, | 0.00) | 0.329 | -0.01 | (-0.02, | 0.00) | 0.402 |

^1^Corrected for 3 comparisons (each trial arm compared to control) for each food group using a Bonferroni-Holm correction.

^2^Lamb and bison.

^3^Plant-based products that aim to simulate meat, for example, Beyond Beef, Impossible Beef, veggie sausage, or mock “chik’n” nuggets.

^4^Mature seeds from the legume family: beans, lentils, or peas.

--, no observations of this food group for this item type.

×, results suppressed in cell sizes < 5 due to high variance.

## Supplemental Table 8. Count of items selected from different food groups, by shopping list task and trial arm: Burrito (N=2,614)

| **Food Group** | **Control**  **(n=683)** | | | **Warning Labels**  **(n=653)** | | | | **Tax**  **(n=640)** | | | | **Combined Warning Labels + Tax**  **(n=638)** | | | |
| --- | --- | --- | --- | --- | --- | --- | --- | --- | --- | --- | --- | --- | --- | --- | --- |
|  | Mean | 95% CI | | Contrast | 95% CI | | *q*^1^ | Contrast | 95% CI | | *q*^1^ | Contrast | 95% CI | | *q*^1^ |
| Unprocessed meat | 0.62 | (0.58, | 0.66) | -0.10 | (-0.16, | -0.04) | **0.002** | -0.13 | (-0.19, | -0.07) | **<0.001** | -0.12 | (-0.18, | -0.05) | **<0.001** |
| Beef | 0.56 | (0.52, | 0.60) | -0.10 | (-0.16, | -0.04) | **0.001** | -0.15 | (-0.21, | -0.09) | **<0.001** | -0.17 | (-0.23, | -0.12) | **<0.001** |
| Pork | -- |  |  | -- |  |  |  | -- |  |  |  | -- |  |  |  |
| Poultry | 0.06 | (0.04, | 0.08) | 0.00 | (-0.02, | 0.03) | 0.838 | 0.01 | (-0.01, | 0.04) | 0.634 | 0.05 | (0.02, | 0.08) | **0.003** |
| Fish & seafood | -- |  |  | -- |  |  |  | -- |  |  |  | -- |  |  |  |
| Other ruminant^2^ | -- |  |  | -- |  |  |  | -- |  |  |  | -- |  |  |  |
|  |  |  |  |  |  |  |  |  |  |  |  |  |  |  |  |
| Processed meat | 0.02 | (0.01, | 0.03) | 0.00 | (-0.02, | 0.01) | 0.652 | -0.01 | (-0.02, | 0.01) | 0.404 | -0.01 | (-0.03, | 0.00) | 0.124 |
| Beef | -- |  |  | -- |  |  |  | -- |  |  |  | -- |  |  |  |
| Pork | 0.02 | (0.01, | 0.03) | 0.00 | (-0.02, | 0.01) | 0.652 | -0.01 | (-0.02, | 0.01) | 0.404 | -0.01 | (-0.03, | 0.00) | 0.124 |
| Poultry | -- |  |  | -- |  |  |  | -- |  |  |  | -- |  |  |  |
| Fish & seafood | -- |  |  | -- |  |  |  | -- |  |  |  | -- |  |  |  |
|  |  |  |  |  |  |  |  |  |  |  |  |  |  |  |  |
| Vegetarian | 0.47 | (0.42, | 0.51) | 0.10 | (0.03, | 0.16) | **0.002** | 0.14 | (0.08, | 0.20) | **<0.001** | 0.15 | (0.09, | 0.21) | **<0.001** |
| Cheese | 0.39 | (0.35, | 0.43) | 0.08 | (0.02, | 0.14) | **0.008** | 0.13 | (0.07, | 0.19) | **<0.001** | 0.13 | (0.07, | 0.19) | **<0.001** |
| Other dairy | -- |  |  | -- |  |  |  | -- |  |  |  | -- |  |  |  |
| Eggs | -- |  |  | -- |  |  |  | -- |  |  |  | -- |  |  |  |
| Tofu | 0.06 | (0.04, | 0.08) | 0.02 | (-0.01, | 0.05) | 0.564 | 0.01 | (-0.02, | 0.03) | 0.677 | 0.02 | (-0.01, | 0.05) | 0.564 |
| Meat mimic^3^ | -- |  |  | -- |  |  |  | -- |  |  |  | -- |  |  |  |
| Pulses^4^ | 0.01 | (0.01, | 0.02) | 0.00 | (-0.01, | 0.01) | >0.999 | 0.01 | (0.00, | 0.03) | 0.431 | 0.00 | (-0.01, | 0.02) | >0.999 |
| Nuts/seeds | -- |  |  | -- |  |  |  | -- |  |  |  | -- |  |  |  |
| Grains & potatoes | -- |  |  | -- |  |  |  | -- |  |  |  | -- |  |  |  |
| Vegetables | -- |  |  | -- |  |  |  | -- |  |  |  | -- |  |  |  |

^1^Corrected for 3 comparisons (each trial arm compared to control) for each food group using a Bonferroni-Holm correction.

^2^Lamb and bison.

^3^Plant-based products that aim to simulate meat, for example, Beyond Beef, Impossible Beef, veggie sausage, or mock “chik’n” nuggets.

^4^Mature seeds from the legume family: beans, lentils, or peas.

--, no observations of this food group for this item type.

## Supplemental Table 9. Count of items selected from different food groups, by shopping list task and trial arm: Burger patties (N=3,045)

| **Food Group** | **Control**  **(n=769)** | | | **Warning Labels**  **(n=783)** | | | | **Tax**  **(n=756)** | | | | **Combined Warning Labels + Tax**  **(n=737)** | | | |
| --- | --- | --- | --- | --- | --- | --- | --- | --- | --- | --- | --- | --- | --- | --- | --- |
|  | Mean | 95% CI | | Contrast | 95% CI | | *q*^1^ | Contrast | 95% CI | | *q*^1^ | Contrast | 95% CI | | *q*^1^ |
| Unprocessed meat | 1.08 | (1.03, | 1.13) | -0.01 | (-0.07, | 0.06) | >0.999 | -0.02 | (-0.09, | 0.05) | >0.999 | -0.08 | (-0.14, | -0.01) | 0.090 |
| Beef | 0.79 | (0.74, | 0.84) | 0.01 | (-0.06, | 0.08) | >0.999 | -0.02 | (-0.09, | 0.05) | >0.999 | -0.11 | (-0.18, | -0.03) | **0.010** |
| Pork | 0.00 | (0.00, | 0.01) | 0.00 | (-0.01, | 0.01) | >0.999 | 0.00 | (-0.01, | 0.00) | >0.999 | 0.00 | (-0.01, | 0.01) | >0.999 |
| Poultry | 0.27 | (0.24, | 0.31) | -0.01 | (-0.06, | 0.04) | >0.999 | 0.01 | (-0.04, | 0.06) | >0.999 | 0.04 | (-0.01, | 0.09) | 0.480 |
| Fish & seafood | -- |  |  | -- |  |  |  | -- |  |  |  | -- |  |  |  |
| Other ruminant^2^ | 0.01 | (0.00, | 0.03) | 0.00 | (-0.02, | 0.01) | >0.999 | -0.01 | (-0.02, | 0.01) | >0.999 | -0.01 | (-0.02, | 0.01) | 0.902 |
|  |  |  |  |  |  |  |  |  |  |  |  |  |  |  |  |
| Processed meat | -- |  |  | -- |  |  |  | -- |  |  |  | -- |  |  |  |
| Beef | -- |  |  | -- |  |  |  | -- |  |  |  | -- |  |  |  |
| Pork | -- |  |  | -- |  |  |  | -- |  |  |  | -- |  |  |  |
| Poultry | -- |  |  | -- |  |  |  | -- |  |  |  | -- |  |  |  |
| Fish & seafood | -- |  |  | -- |  |  |  | -- |  |  |  | -- |  |  |  |
|  |  |  |  |  |  |  |  |  |  |  |  |  |  |  |  |
| Vegetarian | 0.21 | (0.18, | 0.24) | 0.02 | (-0.02, | 0.07) | 0.629 | -0.01 | (-0.05, | 0.04) | 0.746 | 0.05 | (0.00, | 0.10) | 0.143 |
| Cheese | 0.01 | (0.00, | 0.02) | 0.00 | (-0.01, | 0.02) | >0.999 | 0.00 | (-0.01, | 0.01) | >0.999 | 0.00 | (-0.01, | 0.02) | >0.999 |
| Other dairy | -- |  |  | -- |  |  |  | -- |  |  |  | -- |  |  |  |
| Eggs | -- |  |  | -- |  |  |  | -- |  |  |  | -- |  |  |  |
| Tofu | -- |  |  | -- |  |  |  | -- |  |  |  | -- |  |  |  |
| Meat mimic^3^ | 0.19 | (0.16, | 0.22) | 0.01 | (-0.03, | 0.06) | >0.999 | -0.01 | (-0.05, | 0.03) | >0.999 | 0.04 | (-0.01, | 0.08) | 0.398 |
| Pulses^4^ | 0.00 | (0.00, | 0.01) | 0.00 | (0.00, | 0.01) | 0.610 | 0.00 | (0.00, | 0.01) | 0.610 | 0.01 | (0.00, | 0.02) | 0.218 |
| Nuts/seeds | -- |  |  | -- |  |  |  | -- |  |  |  | -- |  |  |  |
| Grains & potatoes | -- |  |  | -- |  |  |  | -- |  |  |  | -- |  |  |  |
| Vegetables | -- |  |  | -- |  |  |  | -- |  |  |  | -- |  |  |  |

^1^Corrected for 3 comparisons (each trial arm compared to control) for each food group using a Bonferroni-Holm correction.

^2^Lamb and bison.

^3^Plant-based products that aim to simulate meat, for example, Beyond Beef, Impossible Beef, veggie sausage, or mock “chik’n” nuggets.

^4^Mature seeds from the legume family: beans, lentils, or peas.

--, no observations of this food group for this item type.

## Supplemental Table 10. Count of items selected from different food groups, by shopping list task and trial arm: Breakfast sausage (N=2,800)

| **Food Group** | **Control**  **(n=707)** | | | **Warning Labels**  **(n=701)** | | | | **Tax**  **(n=709)** | | | | **Combined Warning Labels + Tax**  **(n=683)** | | | |
| --- | --- | --- | --- | --- | --- | --- | --- | --- | --- | --- | --- | --- | --- | --- | --- |
|  | Mean | 95% CI | | Contrast | 95% CI | | *q*^1^ | Contrast | 95% CI | | *q*^1^ | Contrast | 95% CI | | *q*^1^ |
| Unprocessed meat | -- |  |  | -- |  |  |  | -- |  |  |  | -- | -- |  |  |
| Beef | -- |  |  | -- |  |  |  | -- |  |  |  | -- | -- |  |  |
| Pork | -- |  |  | -- |  |  |  | -- |  |  |  | -- | -- |  |  |
| Poultry | -- |  |  | -- |  |  |  | -- |  |  |  | -- | -- |  |  |
| Fish & seafood | -- |  |  | -- |  |  |  | -- |  |  |  | -- | -- |  |  |
| Other ruminant^2^ | -- |  |  | -- |  |  |  | -- |  |  |  | -- | -- |  |  |
|  |  |  |  |  |  |  |  |  |  |  |  |  |  |  |  |
| Processed meat | 1.02 | (0.99, | 1.04) | -0.01 | (-0.04, | 0.02) | >0.999 | 0.00 | (-0.03, | 0.04) | >0.999 | 0.01 | (-0.03, | 0.04) | >0.999 |
| Beef | 0.01 | (0.00, | 0.01) | 0.01 | (0.00, | 0.02) | 0.209 | 0.01 | (0.00, | 0.02) | 0.209 | 0.00 | (-0.01, | 0.01) | 0.700 |
| Pork | 0.88 | (0.85, | 0.91) | -0.06 | (-0.10, | -0.02) | **0.015** | -0.03 | (-0.08, | 0.01) | 0.147 | -0.09 | (-0.14, | -0.04) | **0.001** |
| Poultry | 0.13 | (0.10, | 0.15) | 0.04 | (0.00, | 0.08) | 0.100 | 0.02 | (-0.02, | 0.06) | 0.245 | 0.09 | (0.05, | 0.14) | **<0.001** |
| Fish & seafood | -- |  |  | -- |  |  |  | -- |  |  |  | -- |  |  |  |
|  |  |  |  |  |  |  |  |  |  |  |  |  |  |  |  |
| Vegetarian | 0.03 | (0.02, | 0.04) | 0.00 | (-0.02, | 0.01) | >0.999 | 0.01 | (-0.01, | 0.02) | >0.999 | -0.01 | (-0.02, | 0.01) | >0.999 |
| Cheese | -- |  |  | -- |  |  |  | -- |  |  |  | -- | -- |  |  |
| Other dairy | -- |  |  | -- |  |  |  | -- |  |  |  | -- | -- |  |  |
| Eggs | -- |  |  | -- |  |  |  | -- |  |  |  | -- | -- |  |  |
| Tofu | -- |  |  | -- |  |  |  | -- |  |  |  | -- | -- |  |  |
| Meat mimic^3^ | 0.03 | (0.02, | 0.04) | 0.00 | (-0.02, | 0.01) | >0.999 | 0.01 | (-0.01, | 0.02) | >0.999 | -0.01 | (-0.02, | 0.01) | >0.999 |
| Pulses^4^ | -- |  |  | -- |  |  |  | -- |  |  |  | -- | -- |  |  |
| Nuts/seeds | -- |  |  | -- |  |  |  | -- |  |  |  | -- | -- |  |  |
| Grains & potatoes | -- |  |  | -- |  |  |  | -- |  |  |  | -- | -- |  |  |
| Vegetables | -- |  |  | -- |  |  |  | -- |  |  |  | -- | -- |  |  |

^1^Corrected for 3 comparisons (each trial arm compared to control) for each food group using a Bonferroni-Holm correction.

^2^Lamb and bison.

^3^Plant-based products that aim to simulate meat, for example, Beyond Beef, Impossible Beef, veggie sausage, or mock “chik’n” nuggets.

^4^Mature seeds from the legume family: beans, lentils, or peas.

--, no observations of this food group for this item type.

## Supplemental Table 11. Count of items selected from different food groups, by shopping list task and trial arm: Frozen meal (N=2,769)

| **Food Group** | **Control**  **(n=706)** | | | **Warning Labels**  **(n=697)** | | | | **Tax**  **(n=683)** | | | | **Combined Warning Labels + Tax**  **(n=683)** | | | |
| --- | --- | --- | --- | --- | --- | --- | --- | --- | --- | --- | --- | --- | --- | --- | --- |
|  | Mean | 95% CI | | Contrast | 95% CI | | *q*^1^ | Contrast | 95% CI | | *q*^1^ | Contrast | 95% CI | | *q*^1^ |
| Unprocessed meat | 0.84 | (0.80, | 0.88) | 0.00 | (-0.05, | 0.06) | >0.999 | 0.02 | (-0.03, | 0.08) | >0.999 | -0.01 | (-0.06, | 0.05) | >0.999 |
| Beef | 0.16 | (0.14, | 0.19) | 0.00 | (-0.04, | 0.04) | >0.999 | 0.01 | (-0.04, | 0.05) | >0.999 | -0.03 | (-0.07, | 0.00) | 0.262 |
| Pork | 0.01 | (0.01, | 0.02) | 0.00 | (-0.01, | 0.01) | >0.999 | -0.01 | (-0.02, | 0.00) | 0.644 | 0.00 | (-0.01, | 0.01) | >0.999 |
| Poultry | 0.62 | (0.58, | 0.66) | 0.01 | (-0.06, | 0.07) | >0.999 | 0.03 | (-0.04, | 0.09) | >0.999 | 0.02 | (-0.04, | 0.08) | >0.999 |
| Fish & seafood | 0.04 | (0.02, | 0.05) | -0.01 | (-0.02, | 0.01) | >0.999 | 0.00 | (-0.02, | 0.02) | >0.999 | 0.00 | (-0.02, | 0.02) | >0.999 |
| Other ruminant^2^ | -- |  |  | -- |  |  |  | -- |  |  |  | -- |  |  |  |
|  |  |  |  |  |  |  |  |  |  |  |  |  |  |  |  |
| Processed meat | 0.10 | (0.08, | 0.13) | 0.00 | (-0.04, | 0.04) | 0.995 | -0.02 | (-0.05, | 0.02) | 0.658 | -0.03 | (-0.06, | 0.00) | 0.246 |
| Beef | × |  |  | × |  |  |  | × |  |  |  | × |  |  |  |
| Pork | 0.09 | (0.07, | 0.11) | 0.00 | (-0.03, | 0.04) | 0.818 | -0.01 | (-0.05, | 0.02) | 0.767 | -0.02 | (-0.05, | 0.01) | 0.515 |
| Poultry | 0.01 | (0.00, | 0.01) | 0.00 | (-0.01, | 0.01) | >0.999 | 0.00 | (-0.01, | 0.01) | >0.999 | 0.00 | (-0.01, | 0.00) | 0.813 |
| Fish & seafood | -- |  |  | -- |  |  |  | -- |  |  |  | -- |  |  |  |
|  |  |  |  |  |  |  |  |  |  |  |  |  |  |  |  |
| Vegetarian | 0.16 | (0.13, | 0.19) | 0.02 | (-0.02, | 0.06) | 0.791 | 0.01 | (-0.03, | 0.05) | 0.791 | 0.03 | (-0.01, | 0.07) | 0.471 |
| Cheese | 0.13 | (0.11, | 0.16) | 0.03 | (-0.01, | 0.06) | 0.337 | 0.01 | (-0.02, | 0.05) | 0.437 | 0.03 | (-0.01, | 0.07) | 0.326 |
| Other dairy | 0.00 | (0.00, | 0.01) | 0.00 | (-0.01, | 0.00) | >0.999 | 0.00 | (-0.01, | 0.01) | >0.999 | 0.00 | (-0.01, | 0.00) | 0.987 |
| Eggs | × |  |  | × |  |  |  | × |  |  |  | × |  |  |  |
| Tofu | 0.01 | (0.00, | 0.01) | -0.01 | (-0.01, | 0.00) | 0.075 | 0.00 | (-0.01, | 0.00) | 0.542 | 0.00 | (-0.01, | 0.01) | 0.542 |
| Meat mimic^3^ | × |  |  | × |  |  |  | × |  |  |  | × |  |  |  |
| Pulses^4^ | -- |  |  | -- |  |  |  | -- |  |  |  | -- |  |  |  |
| Nuts/seeds | 0.00 | (0.00, | 0.00) | 0.00 | (0.00, | 0.01) | 0.514 | 0.00 | (0.00, | 0.00) | 0.981 | 0.00 | (0.00, | 0.01) | 0.514 |
| Grains & potatoes | 0.01 | (0.00, | 0.02) | 0.00 | (-0.02, | 0.01) | >0.999 | 0.00 | (-0.01, | 0.01) | >0.999 | 0.00 | (-0.01, | 0.01) | >0.999 |
| Vegetables | × |  |  | × |  |  |  | × |  |  |  | × |  |  |  |

^1^Corrected for 3 comparisons (each trial arm compared to control) for each food group using a Bonferroni-Holm correction.

^2^Lamb and bison.

^3^Plant-based products that aim to simulate meat, for example, Beyond Beef, Impossible Beef, veggie sausage, or mock “chik’n” nuggets.

^4^Mature seeds from the legume family: beans, lentils, or peas.

--, no observations of this food group for this item type.

×, results suppressed in cell sizes < 5 due to high variance.

## Supplemental Table 12. Count of items selected from different food groups, by shopping list task and trial arm: Sandwich and taco fillings (N=3,364)

| **Food Group** | **Control**  **(n=844)** | | | **Warning Labels**  **(n=851)** | | | | **Tax**  **(n=845)** | | | | **Combined Warning Labels + Tax**  **(n=824)** | | | |
| --- | --- | --- | --- | --- | --- | --- | --- | --- | --- | --- | --- | --- | --- | --- | --- |
|  | Mean | 95% CI | | Contrast | 95% CI | | *q*^1^ | Contrast | 95% CI | | *q*^1^ | Contrast | 95% CI | | *q*^1^ |
| Unprocessed meat | 0.51 | (0.47, | 0.56) | -0.02 | (-0.08, | 0.04) | 0.949 | -0.06 | (-0.11, | 0.00) | 0.165 | -0.02 | (-0.08, | 0.04) | 0.949 |
| Beef | 0.18 | (0.15, | 0.20) | -0.02 | (-0.05, | 0.02) | 0.431 | -0.04 | (-0.07, | 0.00) | 0.186 | -0.03 | (-0.07, | 0.00) | 0.186 |
| Pork | 0.02 | (0.01, | 0.03) | 0.00 | (-0.02, | 0.01) | 0.714 | -0.01 | (-0.02, | 0.00) | 0.243 | -0.01 | (-0.02, | 0.00) | 0.282 |
| Poultry | 0.28 | (0.25, | 0.31) | 0.00 | (-0.05, | 0.05) | >0.999 | 0.01 | (-0.04, | 0.05) | >0.999 | 0.03 | (-0.02, | 0.08) | 0.563 |
| Fish & seafood | 0.04 | (0.02, | 0.05) | 0.00 | (-0.02, | 0.02) | 0.890 | -0.02 | (-0.04, | 0.00) | 0.190 | -0.01 | (-0.03, | 0.01) | 0.600 |
| Other ruminant^2^ | -- |  |  | -- |  |  |  | -- |  |  |  | -- |  |  |  |
|  |  |  |  |  |  |  |  |  |  |  |  |  |  |  |  |
| Processed meat | 0.76 | (0.71, | 0.80) | -0.01 | (-0.08, | 0.05) | >0.999 | -0.02 | (-0.09, | 0.04) | >0.999 | -0.04 | (-0.10, | 0.02) | 0.492 |
| Beef | 0.08 | (0.06, | 0.10) | -0.01 | (-0.04, | 0.01) | 0.800 | 0.01 | (-0.03, | 0.04) | 0.800 | -0.02 | (-0.05, | 0.01) | 0.414 |
| Pork | 0.35 | (0.31, | 0.39) | -0.01 | (-0.06, | 0.05) | 0.784 | -0.06 | (-0.11, | -0.01) | 0.051 | -0.08 | (-0.13, | -0.03) | **0.005** |
| Poultry | 0.32 | (0.29, | 0.36) | 0.01 | (-0.04, | 0.05) | 0.823 | 0.03 | (-0.02, | 0.08) | 0.357 | 0.06 | (0.01, | 0.11) | 0.063 |
| Fish & seafood | × |  |  | × |  |  |  | × |  |  |  | × |  |  |  |
|  |  |  |  |  |  |  |  |  |  |  |  |  |  |  |  |
| Vegetarian | 0.71 | (0.64, | 0.77) | 0.01 | (-0.08, | 0.10) | 0.882 | 0.04 | (-0.05, | 0.14) | 0.882 | 0.05 | (-0.04, | 0.15) | 0.882 |
| Cheese | 0.06 | (0.04, | 0.08) | 0.03 | (-0.01, | 0.06) | 0.393 | 0.01 | (-0.02, | 0.04) | >0.999 | -0.01 | (-0.03, | 0.02) | >0.999 |
| Other dairy | 0.00 | (0.00, | 0.01) | 0.00 | (-0.01, | 0.01) | >0.999 | 0.00 | (-0.01, | 0.01) | >0.999 | 0.00 | (-0.01, | 0.01) | >0.999 |
| Eggs | 0.04 | (0.02, | 0.05) | 0.00 | (-0.02, | 0.02) | >0.999 | 0.00 | (-0.02, | 0.02) | >0.999 | 0.02 | (-0.01, | 0.06) | 0.429 |
| Tofu | -- |  |  | -- |  |  |  | -- |  |  |  | -- |  |  |  |
| Meat mimic^3^ | 0.00 | (0.00, | 0.01) | 0.00 | (-0.01, | 0.00) | 0.471 | 0.00 | (0.00, | 0.00) | >0.999 | 0.00 | (0.00, | 0.01) | >0.999 |
| Pulses^4^ | 0.22 | (0.19, | 0.25) | 0.02 | (-0.03, | 0.06) | 0.472 | 0.05 | (0.00, | 0.09) | 0.117 | 0.04 | (-0.01, | 0.08) | 0.265 |
| Nuts/seeds | 0.23 | (0.21, | 0.26) | 0.03 | (-0.02, | 0.07) | 0.616 | 0.03 | (-0.01, | 0.07) | 0.616 | 0.02 | (-0.02, | 0.06) | 0.616 |
| Grains & potatoes | 0.01 | (0.00, | 0.02) | 0.00 | (-0.01, | 0.01) | >0.999 | 0.00 | (-0.01, | 0.01) | >0.999 | 0.00 | (-0.01, | 0.00) | 0.906 |
| Vegetables | 0.14 | (0.09, | 0.18) | -0.06 | (-0.11, | -0.01) | 0.052 | -0.04 | (-0.09, | 0.01) | 0.326 | -0.02 | (-0.07, | 0.04) | 0.549 |

^1^Corrected for 3 comparisons (each trial arm compared to control) for each food group using a Bonferroni-Holm correction.

^2^Lamb and bison.

^3^Plant-based products that aim to simulate meat, for example, Beyond Beef, Impossible Beef, veggie sausage, or mock “chik’n” nuggets.

^4^Mature seeds from the legume family: beans, lentils, or peas.

--, no observations of this food group for this item type.

×, results suppressed in cell sizes < 5 due to high variance.
